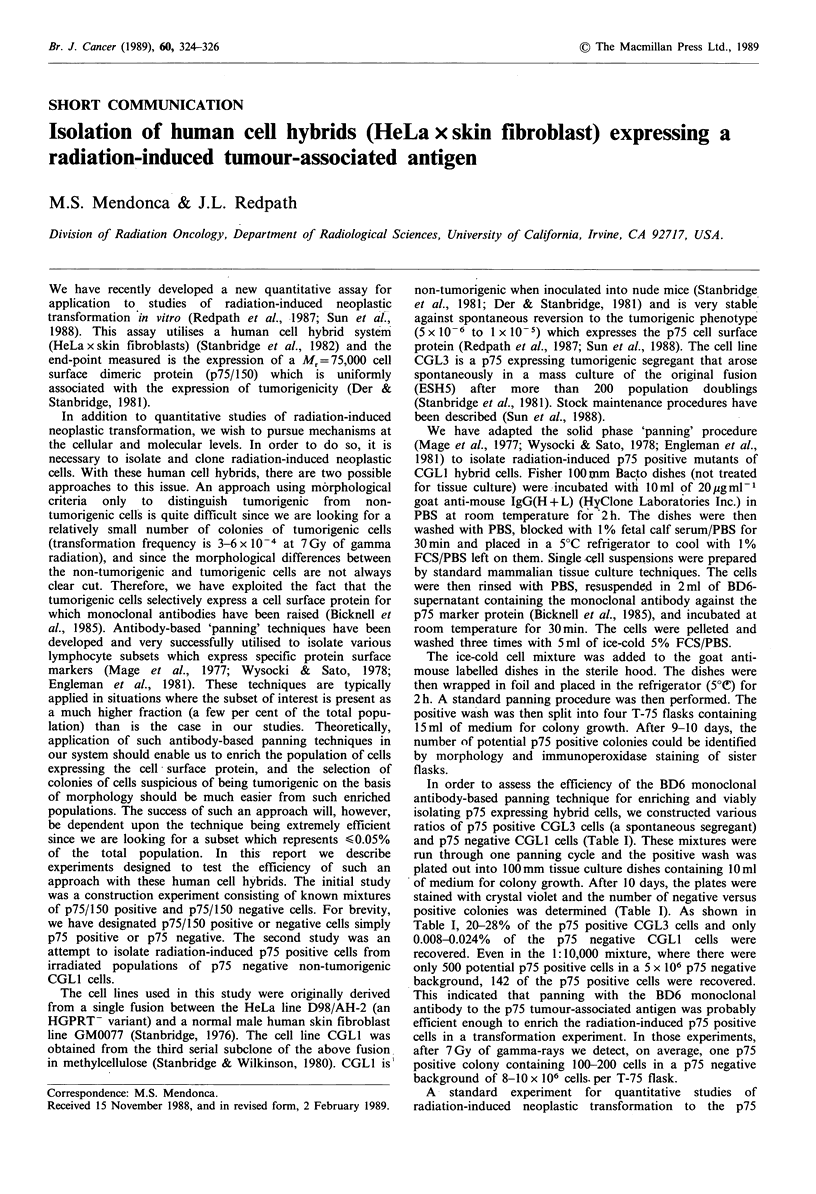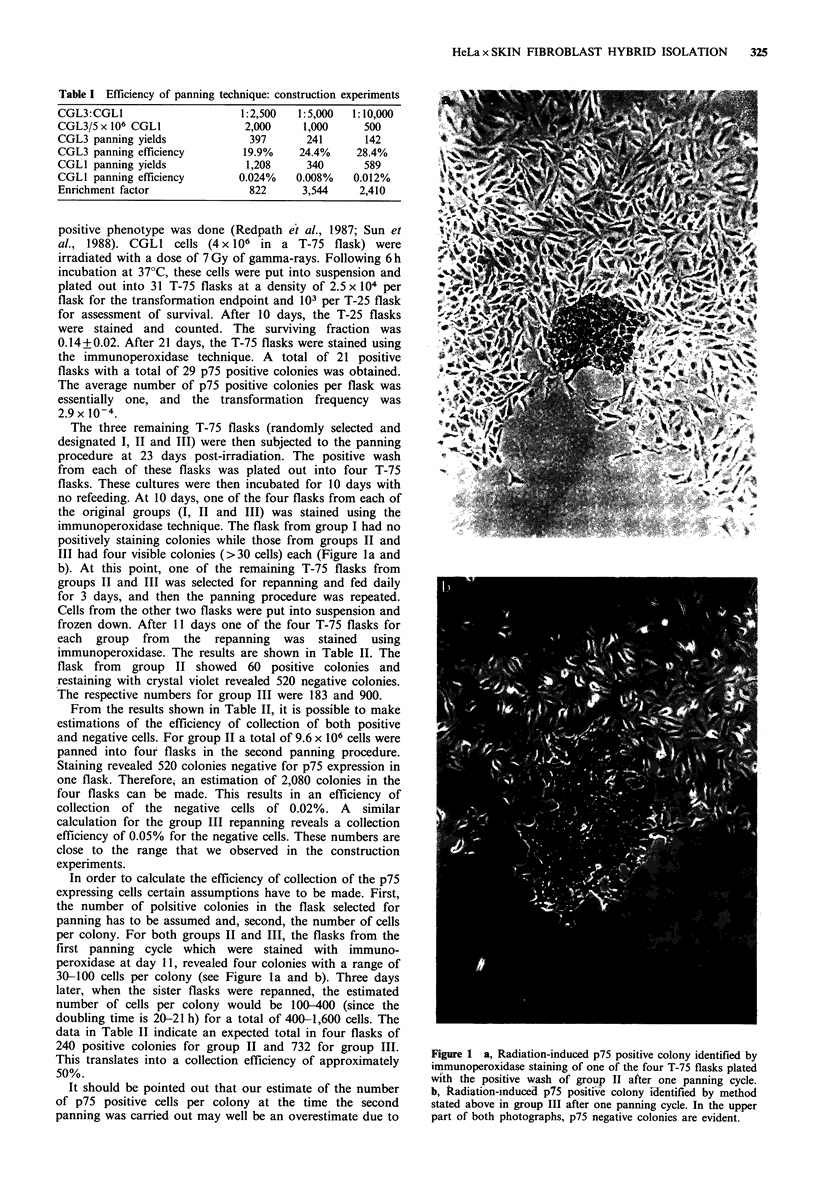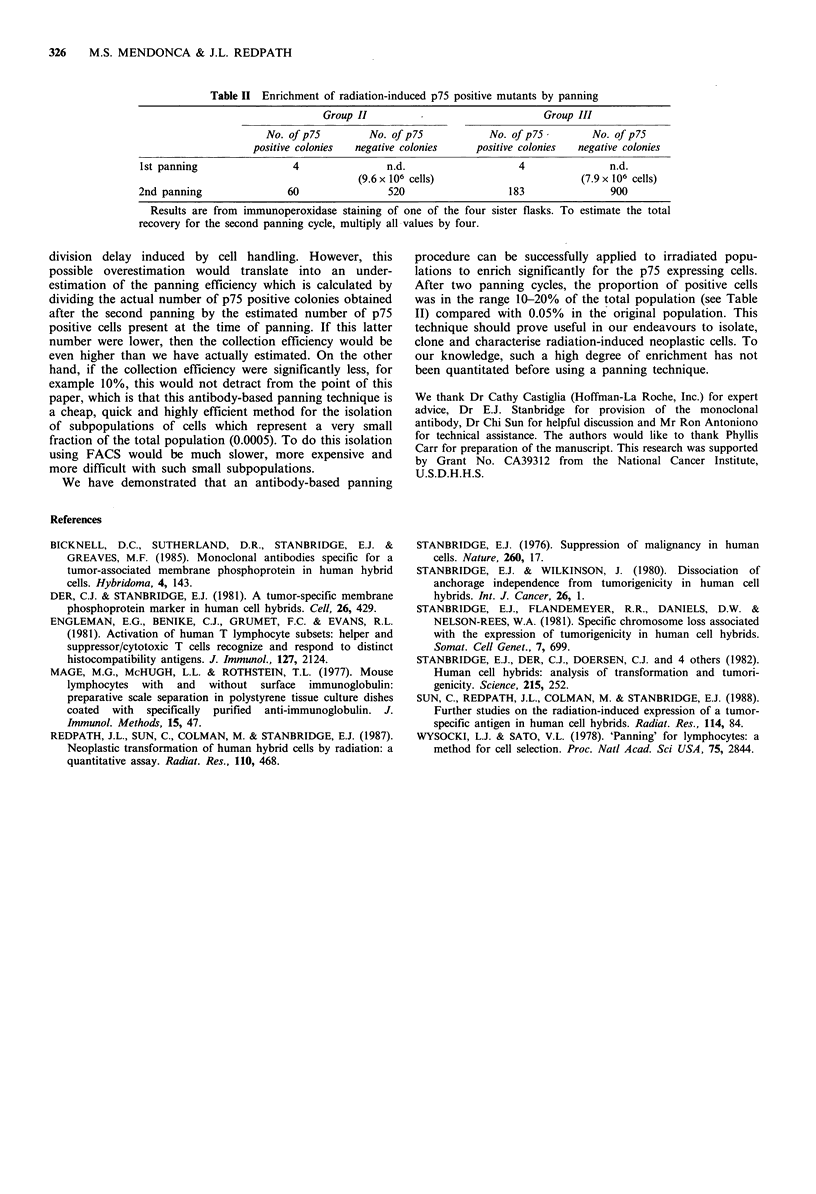# Isolation of human cell hybrids (HeLa x skin fibroblast) expressing a radiation-induced tumour-associated antigen.

**DOI:** 10.1038/bjc.1989.279

**Published:** 1989-09

**Authors:** M. S. Mendonca, J. L. Redpath

**Affiliations:** Department of Radiological Sciences, University of California, Irvine 92717.

## Abstract

**Images:**


*Br. J. Cancer* (1989), **60**, 324–326 © The Macmillan Press Ltd., 1989

SHORT COMMUNICATION

### Isolation of human cell hybrids (HeLa × skin fibroblast) expressing a radiation-induced tumour-associated antigen

M.S. Mendonca & J.L. Redpath

*Division of Radiation Oncology, Department of Radiological Sciences, University of California, Irvine, CA 92717, USA.*

We have recently developed a new quantitative assay for application to studies of radiation-induced neoplastic transformation *in vitro* (Redpath *et al.*, 1987; Sun *et al.*, 1988). This assay utilises a human cell hybrid system (HeLa × skin fibroblasts) (Stanbridge *et al.*, 1982) and the end-point measured is the expression of a $M_r = 75{,}000$ cell surface dimeric protein (p75/150) which is uniformly associated with the expression of tumorigenicity (Der & Stanbridge, 1981).

In addition to quantitative studies of radiation-induced neoplastic transformation, we wish to pursue mechanisms at the cellular and molecular levels. In order to do so, it is necessary to isolate and clone radiation-induced neoplastic cells. With these human cell hybrids, there are two possible approaches to this issue. An approach using morphological criteria only to distinguish tumorigenic from non-tumorigenic cells is quite difficult since we are looking for a relatively small number of colonies of tumorigenic cells (transformation frequency is $3–6 \times 10^{-4}$ at 7 Gy of gamma radiation), and since the morphological differences between the non-tumorigenic and tumorigenic cells are not always clear cut. Therefore, we have exploited the fact that the tumorigenic cells selectively express a cell surface protein for which monoclonal antibodies have been raised (Bicknell *et al.*, 1985). Antibody-based 'panning' techniques have been developed and very successfully utilised to isolate various lymphocyte subsets which express specific protein surface markers (Mage *et al.*, 1977; Wysocki & Sato, 1978; Engleman *et al.*, 1981). These techniques are typically applied in situations where the subset of interest is present as a much higher fraction (a few per cent of the total population) than is the case in our studies. Theoretically, application of such antibody-based panning techniques in our system should enable us to enrich the population of cells expressing the cell surface protein, and the selection of colonies of cells suspicious of being tumorigenic on the basis of morphology should be much easier from such enriched populations. The success of such an approach will, however, be dependent upon the technique being extremely efficient since we are looking for a subset which represents ⩽0.05% of the total population. In this report we describe experiments designed to test the efficiency of such an approach with these human cell hybrids. The initial study was a construction experiment consisting of known mixtures of p75/150 positive and p75/150 negative cells. For brevity, we have designated p75/150 positive or negative cells simply p75 positive or p75 negative. The second study was an attempt to isolate radiation-induced p75 positive cells from irradiated populations of p75 negative non-tumorigenic CGL1 cells.

The cell lines used in this study were originally derived from a single fusion between the HeLa line D98/AH-2 (an $HGPRT^-$ variant) and a normal male human skin fibroblast line GM0077 (Stanbridge, 1976). The cell line CGL1 was obtained from the third serial subclone of the above fusion in methylcellulose (Stanbridge & Wilkinson, 1980). CGL1 is non-tumorigenic when inoculated into nude mice (Stanbridge *et al.*, 1981; Der & Stanbridge, 1981) and is very stable against spontaneous reversion to the tumorigenic phenotype ($5 \times 10^{-6}$ to $1 \times 10^{-5}$) which expresses the p75 cell surface protein (Redpath *et al.*, 1987; Sun *et al.*, 1988). The cell line CGL3 is a p75 expressing tumorigenic segregant that arose spontaneously in a mass culture of the original fusion (ESH5) after more than 200 population doublings (Stanbridge *et al.*, 1981). Stock maintenance procedures have been described (Sun *et al.*, 1988).

We have adapted the solid phase 'panning' procedure (Mage *et al.*, 1977; Wysocki & Sato, 1978; Engleman *et al.*, 1981) to isolate radiation-induced p75 positive mutants of CGL1 hybrid cells. Fisher 100 mm Bacto dishes (not treated for tissue culture) were incubated with 10 ml of $20\,\mu g\,ml^{-1}$ goat anti-mouse IgG(H + L) (HyClone Laboratories Inc.) in PBS at room temperature for 2 h. The dishes were then washed with PBS, blocked with 1% fetal calf serum/PBS for 30 min and placed in a 5°C refrigerator to cool with 1% FCS/PBS left on them. Single cell suspensions were prepared by standard mammalian tissue culture techniques. The cells were then rinsed with PBS, resuspended in 2 ml of BD6-supernatant containing the monoclonal antibody against the p75 marker protein (Bicknell *et al.*, 1985), and incubated at room temperature for 30 min. The cells were pelleted and washed three times with 5 ml of ice-cold 5% FCS/PBS.

The ice-cold cell mixture was added to the goat anti-mouse labelled dishes in the sterile hood. The dishes were then wrapped in foil and placed in the refrigerator (5°C) for 2 h. A standard panning procedure was then performed. The positive wash was then split into four T-75 flasks containing 15 ml of medium for colony growth. After 9–10 days, the number of potential p75 positive colonies could be identified by morphology and immunoperoxidase staining of sister flasks.

In order to assess the efficiency of the BD6 monoclonal antibody-based panning technique for enriching and viably isolating p75 expressing hybrid cells, we constructed various ratios of p75 positive CGL3 cells (a spontaneous segregant) and p75 negative CGL1 cells (Table I). These mixtures were run through one panning cycle and the positive wash was plated out into 100 mm tissue culture dishes containing 10 ml of medium for colony growth. After 10 days, the plates were stained with crystal violet and the number of negative versus positive colonies was determined (Table I). As shown in Table I, 20–28% of the p75 positive CGL3 cells and only 0.008–0.024% of the p75 negative CGL1 cells were recovered. Even in the 1:10,000 mixture, where there were only 500 potential p75 positive cells in a $5 \times 10^6$ p75 negative background, 142 of the p75 positive cells were recovered. This indicated that panning with the BD6 monoclonal antibody to the p75 tumour-associated antigen was probably efficient enough to enrich the radiation-induced p75 positive cells in a transformation experiment. In those experiments, after 7 Gy of gamma-rays we detect, on average, one p75 positive colony containing 100–200 cells in a p75 negative background of $8–10 \times 10^6$ cells per T-75 flask.

A standard experiment for quantitative studies of radiation-induced neoplastic transformation to the p75

Correspondence: M.S. Mendonca.
Received 15 November 1988, and in revised form, 2 February 1989.

**Table I** Efficiency of panning technique: construction experiments

| | | | |
|---|---|---|---|
| CGL3:CGL1 | 1:2,500 | 1:5,000 | 1:10,000 |
| CGL3/5 x $10^6$ CGL1 | 2,000 | 1,000 | 500 |
| CGL3 panning yields | 397 | 241 | 142 |
| CGL3 panning efficiency | 19.9% | 24.4% | 28.4% |
| CGL1 panning yields | 1,208 | 340 | 589 |
| CGL1 panning efficiency | 0.024% | 0.008% | 0.012% |
| Enrichment factor | 822 | 3,544 | 2,410 |

positive phenotype was done (Redpath *et al.*, 1987; Sun *et al.*, 1988). CGL1 cells (4 x $10^6$ in a T-75 flask) were irradiated with a dose of 7 Gy of gamma-rays. Following 6 h incubation at 37°C, these cells were put into suspension and plated out into 31 T-75 flasks at a density of 2.5 x $10^4$ per flask for the transformation endpoint and $10^3$ per T-25 flask for assessment of survival. After 10 days, the T-25 flasks were stained and counted. The surviving fraction was 0.14$\pm$0.02. After 21 days, the T-75 flasks were stained using the immunoperoxidase technique. A total of 21 positive flasks with a total of 29 p75 positive colonies was obtained. The average number of p75 positive colonies per flask was essentially one, and the transformation frequency was 2.9 x $10^{-4}$.

The three remaining T-75 flasks (randomly selected and designated I, II and III) were then subjected to the panning procedure at 23 days post-irradiation. The positive wash from each of these flasks was plated out into four T-75 flasks. These cultures were then incubated for 10 days with no refeeding. At 10 days, one of the four flasks from each of the original groups (I, II and III) was stained using the immunoperoxidase technique. The flask from group I had no positively staining colonies while those from groups II and III had four visible colonies (>30 cells) each (Figure 1a and b). At this point, one of the remaining T-75 flasks from groups II and III was selected for repanning and fed daily for 3 days, and then the panning procedure was repeated. Cells from the other two flasks were put into suspension and frozen down. After 11 days one of the four T-75 flasks for each group from the repanning was stained using immunoperoxidase. The results are shown in Table II. The flask from group II showed 60 positive colonies and restaining with crystal violet revealed 520 negative colonies. The respective numbers for group III were 183 and 900.

From the results shown in Table II, it is possible to make estimations of the efficiency of collection of both positive and negative cells. For group II a total of 9.6 x $10^6$ cells were panned into four flasks in the second panning procedure. Staining revealed 520 colonies negative for p75 expression in one flask. Therefore, an estimation of 2,080 colonies in the four flasks can be made. This results in an efficiency of collection of the negative cells of 0.02%. A similar calculation for the group III repanning reveals a collection efficiency of 0.05% for the negative cells. These numbers are close to the range that we observed in the construction experiments.

In order to calculate the efficiency of collection of the p75 expressing cells certain assumptions have to be made. First, the number of polsitive colonies in the flask selected for panning has to be assumed and, second, the number of cells per colony. For both groups II and III, the flasks from the first panning cycle which were stained with immunoperoxidase at day 11, revealed four colonies with a range of 30–100 cells per colony (see Figure 1a and b). Three days later, when the sister flasks were repanned, the estimated number of cells per colony would be 100–400 (since the doubling time is 20–21 h) for a total of 400–1,600 cells. The data in Table II indicate an expected total in four flasks of 240 positive colonies for group II and 732 for group III. This translates into a collection efficiency of approximately 50%.

It should be pointed out that our estimate of the number of p75 positive cells per colony at the time the second panning was carried out may well be an overestimate due to

**Figure 1** **a**, Radiation-induced p75 positive colony identified by immunoperoxidase staining of one of the four T-75 flasks plated with the positive wash of group II after one panning cycle. **b**, Radiation-induced p75 positive colony identified by method stated above in group III after one panning cycle. In the upper part of both photographs, p75 negative colonies are evident.

**Table II** Enrichment of radiation-induced p75 positive mutants by panning

| | *Group II* | | *Group III* | |
|---|---|---|---|---|
| | *No. of p75 positive colonies* | *No. of p75 negative colonies* | *No. of p75 positive colonies* | *No. of p75 negative colonies* |
| 1st panning | 4 | n.d. ($9.6 \times 10^6$ cells) | 4 | n.d. ($7.9 \times 10^6$ cells) |
| 2nd panning | 60 | 520 | 183 | 900 |

Results are from immunoperoxidase staining of one of the four sister flasks. To estimate the total recovery for the second panning cycle, multiply all values by four.

division delay induced by cell handling. However, this possible overestimation would translate into an underestimation of the panning efficiency which is calculated by dividing the actual number of p75 positive colonies obtained after the second panning by the estimated number of p75 positive cells present at the time of panning. If this latter number were lower, then the collection efficiency would be even higher than we have actually estimated. On the other hand, if the collection efficiency were significantly less, for example 10%, this would not detract from the point of this paper, which is that this antibody-based panning technique is a cheap, quick and highly efficient method for the isolation of subpopulations of cells which represent a very small fraction of the total population (0.0005). To do this isolation using FACS would be much slower, more expensive and more difficult with such small subpopulations.

We have demonstrated that an antibody-based panning procedure can be successfully applied to irradiated populations to enrich significantly for the p75 expressing cells. After two panning cycles, the proportion of positive cells was in the range 10–20% of the total population (see Table II) compared with 0.05% in the original population. This technique should prove useful in our endeavours to isolate, clone and characterise radiation-induced neoplastic cells. To our knowledge, such a high degree of enrichment has not been quantitated before using a panning technique.

We thank Dr Cathy Castiglia (Hoffman-La Roche, Inc.) for expert advice, Dr E.J. Stanbridge for provision of the monoclonal antibody, Dr Chi Sun for helpful discussion and Mr Ron Antoniono for technical assistance. The authors would like to thank Phyllis Carr for preparation of the manuscript. This research was supported by Grant No. CA39312 from the National Cancer Institute, U.S.D.H.H.S.

### References

BICKNELL, D.C., SUTHERLAND, D.R., STANBRIDGE, E.J. & GREAVES, M.F. (1985). Monoclonal antibodies specific for a tumor-associated membrane phosphoprotein in human hybrid cells. *Hybridoma*, **4**, 143.

DER, C.J. & STANBRIDGE, E.J. (1981). A tumor-specific membrane phosphoprotein marker in human cell hybrids. *Cell*, **26**, 429.

ENGLEMAN, E.G., BENIKE, C.J., GRUMET, F.C. & EVANS, R.L. (1981). Activation of human T lymphocyte subsets: helper and suppressor/cytotoxic T cells recognize and respond to distinct histocompatibility antigens. *J. Immunol.*, **127**, 2124.

MAGE, M.G., McHUGH, L.L. & ROTHSTEIN, T.L. (1977). Mouse lymphocytes with and without surface immunoglobulin: preparative scale separation in polystyrene tissue culture dishes coated with specifically purified anti-immunoglobulin. *J. Immunol. Methods*, **15**, 47.

REDPATH, J.L., SUN, C., COLMAN, M. & STANBRIDGE, E.J. (1987). Neoplastic transformation of human hybrid cells by radiation: a quantitative assay. *Radiat. Res.*, **110**, 468.

STANBRIDGE, E.J. (1976). Suppression of malignancy in human cells. *Nature*, **260**, 17.

STANBRIDGE, E.J. & WILKINSON, J. (1980). Dissociation of anchorage independence from tumorigenicity in human cell hybrids. *Int. J. Cancer*, **26**, 1.

STANBRIDGE, E.J., FLANDEMEYER, R.R., DANIELS, D.W. & NELSON-REES, W.A. (1981). Specific chromosome loss associated with the expression of tumorigenicity in human cell hybrids. *Somat. Cell Genet.*, **7**, 699.

STANBRIDGE, E.J., DER, C.J., DOERSEN, C.J. and 4 others (1982). Human cell hybrids: analysis of transformation and tumorigenicity. *Science*, **215**, 252.

SUN, C., REDPATH, J.L., COLMAN, M. & STANBRIDGE, E.J. (1988). Further studies on the radiation-induced expression of a tumor-specific antigen in human cell hybrids. *Radiat. Res.*, **114**, 84.

WYSOCKI, L.J. & SATO, V.L. (1978). 'Panning' for lymphocytes: a method for cell selection. *Proc. Natl Acad. Sci USA*, **75**, 2844.